# Perspectives of Older Adults on Assistive Technology: Qualitative Study

**DOI:** 10.2196/74214

**Published:** 2025-11-12

**Authors:** Mirou Jaana, Maude Lévesque Ryan, Haitham Tamim, Edward Riachy, Guy Paré

**Affiliations:** 1 Telfer School of Management, University of Ottawa, 55 Laurier Ave E, Ottawa, ON, K1N 9B9, Canada, 1 (613) 562 5800 ext 3400; 2 Department of public health-infectious diseases, Centre intégré de santé et de services sociaux de Chaudière-Appalaches, Sainte-Marie, QC, Canada; 3 School of Business and Hospitality, Algonquin College, Ottawa, ON, Canada; 4 Department of Information Technology, HEC Montréal, Montréal, QC, Canada

**Keywords:** aging, assistive technology, telehealth, telemonitoring, self-monitoring, fall detection, patient acceptance, technology adoption, qualitative research, focus groups, interviews

## Abstract

**Background:**

The aging population presents challenges for health care systems. Assistive technologies (ATs), such as telemonitoring, fall detection, and self-monitoring devices, offer potential solutions to support older adults and their care. However, successful implementation relies on their acceptance, which remains poorly understood, particularly among nonusers.

**Objective:**

This study aimed to explore older adults’ perceptions of ATs, including perceived benefits, adoption barriers, and factors influencing willingness to use these technologies.

**Methods:**

A qualitative study was conducted with 31 participants (aged ≥65 years) with varying levels of health and care needs. Data were collected through 6 focus groups and 6 in-depth interviews and then analyzed thematically using NVivo software.

**Results:**

A total of 7 themes emerged: (1) limited familiarity, with greater recognition of fall detection and self-monitoring devices compared to telemonitoring; (2) perceived benefits, including safety, independence, and chronic disease management; (3) key concerns, including usability, cost, reliability, privacy, and psychological impacts; (4) suggested improvements, including user-friendly designs and training programs; (5) contextual influences identified with independent older adults perceiving greater utility; (6) strategies for ATs’ promotion proposed, such as media campaigns, government subsidies, and health care endorsements; and (7) overall willingness to adopt ATs, driven by perceived need, social and health care influence, and ease of use.

**Conclusions:**

Although ATs offer clear benefits, adoption remains limited due to usability, cost, and psychological concerns. Improving accessibility, training, and integration into traditional health care service delivery may facilitate acceptance and use. Future research should focus on inclusive designs and policy interventions to maximize ATs' potential in aging populations.

## Introduction

### Background

The global population is aging rapidly, posing challenges to health care systems. In Canada, adults aged ≥65 years comprised 14% of the population in 2009 and are expected to reach 23% by 2030 [1]. Providing them with proper care and enabling them to age comfortably at home is crucial, with significant societal and economic implications [2]. Effective management of chronic conditions and health deterioration can improve well-being [3] while reducing costs related to institutional care [4-6].

Assistive technologies (ATs) offer solutions to support older adults’ health and independence [3-5,7-9]. Telemonitoring enhances quality of life and security, particularly for those with chronic diseases [4,5,10-15]. Fall detection devices help prevent injuries and allow for timely interventions, reducing long hospital stays [5,8,16]. Self-monitoring devices promote healthier lifestyles by tracking movement and activity levels, providing real-time insights for better care [3,5,17,18].

Nevertheless, concerns remain about older adults’ acceptance of these technologies [3,6-8,19-22], as much of the research focuses primarily on technological innovations or those using these technologies. For ATs to be effective, older adults must be inclined to use them, which depends on acceptance [7,22], motivation, and understanding of their use [8]. Placing them at the center of research ensures that these technologies are practical and accessible [23-31]. Nevertheless, studying older adults, particularly the more vulnerable subgroup, is challenging, leading to knowledge gaps, particularly regarding the perspectives of nonusers [4,32,33]. Prior studies have mostly focused on existing AT users [26,34-37] or specific health conditions such as chronic obstructive pulmonary disease [38-40], hypertension [36,41], heart disease [39] and diabetes [36,42], limiting insights into the broader older population’s views [43].

This study aimed to address this gap by investigating how older adults, with various health statuses and experiences, perceive and approach AT types—telemonitoring, fall detection technology, and self-monitoring devices.

### Literature Review

Technology-enabled health care is an umbrella term referring to technologies (eg, information and communication, mobile technologies, etc.) that are designed and tailored to provide care, improve the quality of health services, and promote well-being [44]. eHealth is another term that is used in reference to information and communication technologies used for health and encompasses a variety of mobile health apps [45,46] and internet use for health services and information delivery [47,48]. Digital ATs (eg, communication systems, ambient technologies or monitoring devices, mobile apps, etc.) represent the products or systems used to “help maintain or improve an individual’s functioning related to cognition, communication, hearing, mobility, self-care, and vision, thus enabling their health, well-being, inclusion, and participation” [49].

ATs present benefits to older adults’ care, given their ability to support health care professionals’ work (eg, by providing older adults with assistance with activities of daily living), assist older adults in completing tasks that would otherwise not be possible (eg, mobility), and enable older adults to feel safe while independently living at home [50]. In a recent systematic review of randomized controlled trials, ATs were found to be effective for personal disease management (eg, statistically significant positive effects on self-care and health-related quality of life), medication management (eg, statistically significant improvements in medication adherence and a reduction in the number of missed doses), and vision (eg, statistically significant improvements in visual functions with the use of ATs) in older adults [51]. Prior studies have also highlighted other benefits for older adults, including reduced falls [52], more autonomy and independence [53], improved social connection and engagement, and enhanced well-being [54] when using ATs.

Despite the demonstrated benefits and effectiveness of these technologies, older adults’ use of ATs remains low, particularly among those with multimorbidity who may benefit most from their use [51]. Previous studies have investigated older adults’ acceptance of ATs. For example, Shin et al [55] assessed South Korean older adults’ acceptance of ATs using the Senior Technology Acceptance Model. They found that gerontechnology self-efficacy, health contexts and abilities, attitudinal factors, perceived usefulness, and perceived ease of use statistically affected older adults’ intention to use ATs [55]. A recently published meta-analysis reviewed the literature that used the Technology Acceptance Model and Unified Theory of Acceptance and Use of Technology (UTAUT) to assess older adults’ acceptance of technology [56]. They found that perceived usefulness, social influence, and ease of use all significantly impact older adults’ acceptance of ATs, although this relationship is moderated by the type of AT considered [56], with high heterogeneity across the studies considered. As a result, the authors recommended that future research explore the variations in acceptance patterns across different contexts and technologies to lead to a more comprehensive understanding of older adults’ AT acceptance [56].

While the existing literature using the TAM, Senior Technology Acceptance Model (STAM), UTAUT, and other behavioral theories has contributed to our understanding of older adults’ technology acceptance, there have been calls in the literature for more qualitative research that explores dimensions beyond those included in existing theories [57]. For example, Felber et al [57] discussed that research often quantitatively validates these theories, without consideration of additional issues that are relevant to older adults (eg, loneliness, the fear of ATs replacing human care, etc.). As such, qualitative research would be appropriate to allow additional factors that may influence acceptance to emerge from the data [58]. They are best suited for studies aiming to understand the reasons behind why an older adult may or may not accept a technology and for providing a more detailed understanding of the nuances that are often missed when quantitative approaches are used [57,58]. This study provides new insights into this area of research by exploring how older adults in long-term care (LTC) environments perceive and approach 3 different AT types (ie, telemonitoring, fall detection technology, and self-monitoring devices) using qualitative methods.

## Methods

### Study Setting

The study was conducted at Perley Health, Ottawa’s largest LTC home. Perley Health is a comprehensive community of care that is home to more than 600 seniors and veterans across 450 LTC beds and 139 independent living apartments. It is also a center for research, education, and clinical innovation and offers many programs and services to residents, tenants, and people from across the region. As part of a pilot project from 2018 to 2021, Perley Health had a subacute unit for frail elderly (SAFE), which was a 20-bed specialty care unit that cared for patients recovering from surgery, illness, or other acute health events, who no longer needed to be in hospital but were not ready to go home. Patients typically stayed on this unit for 2 to 3 weeks to access medical and rehabilitative supports and then returned to their place of residence in the community.

Recruitment began in late October 2018 and concluded in April 2019. The process was adapted to participants’ autonomy levels. LTC and SAFE residents required multiple visits to build trust, address hesitations, and encourage engagement. For ILA residents, recruitment involved flyers and group sessions to present the study and promote participation.

### Study Design and Population

We used a qualitative research design to capture diverse perspectives of older adults with varying levels of independence and care needs and residing in different environments. This approach combined focus groups, encouraging discussion and interaction [59,60], with in-depth interviews for a deeper exploration of individual experiences [60].

Participants aged ≥65 years were selected through purposive sampling to ensure diversity in health status, living arrangements, and technology familiarity. Inclusion criteria required cognitive capability to be able to provide informed consent, which was usually assessed using the Cognitive Performance Scale [61]. Individuals with moderate to severe impairment were excluded because of their inability to participate.

### Data Collection and Analysis

We conducted 6 focus groups (4‐6 participants each), grouping individuals by autonomy level (eg, ILA vs LTC). Discussions covered experiences with ATs, perceived benefits, challenges, usefulness, willingness to adopt the technologies, and suggestions for improvement. As part of the original study design, we also conducted 6 in-depth interviews with participants who were unable to attend group discussions. These interviews provided deeper insights into specific themes and ensured a balanced representation of opinions.

The 3 ATs explored in this study were selected for their relevance to aging in place and their practical applicability for older adults in institutional or community care settings. Telemonitoring refers to remote systems that track vital signs or health conditions and transmit data to health care providers [62]. Fall detection devices are typically wearable sensors that automatically alert caregivers or emergency services when a fall is detected [63]. Self-monitoring tools, such as GPS-enabled trackers or activity monitors, allow users and family members to track location or daily activity for enhanced safety [64].

Other digital health tools, such as mobile health apps, virtual reality, or digital twin systems, were not included, as they were considered less familiar or accessible to the study population. The selected technologies reflect those most commonly promoted for older adult safety, autonomy, and chronic condition support in residential and home-based care contexts.

To facilitate discussion, participants watched short, brand-neutral, publicly available videos (1‐2 min each) introducing each technology. These videos emphasized core features, use cases, and benefits while reducing cognitive effort and improving comprehension. Following each video, a series of open-ended and targeted questions were posed using a semistructured discussion guide. These questions were designed to explore perceived benefits (eg, timely interventions and independence), concerns (eg, cost, usability, privacy, and reliability), and potential adoption triggers (eg, health status changes and physician recommendation). Participants were specifically asked whether they believed the technology could enable timely emergency intervention and what limitations or barriers they perceived. The guide also differentiated between those who had previously used the technology and those who had not, allowing for comparison between experienced users and nonusers. Questions were adapted to each device type (telemonitoring, fall detection, and self-tracking) and to the participant’s care setting.

Focus groups and interviews lasted up to 1 hour and were audio recorded, with written notes capturing nonverbal cues and context. We transcribed the audio recordings verbatim and analyzed them thematically using NVivo (QSR International, Melbourne, Australia) software. Thematic analysis followed a partially inductive approach: although the interview guide informed the initial structure, codes and subthemes emerged directly from participant narratives. After subclassifying the main themes, a sample of 100 randomly selected coded passages was independently reviewed by 2 research assistants. Discrepancies in interpretation were discussed and resolved collaboratively to enhance coding consistency and validity. To preserve confidentiality and adhere to ethical requirements, participant quotes were not linked to individual identifiers or care settings. As such, quotes are presented without attribution beyond their thematic grouping.

### Ethical Considerations

Ethics approval was granted from the University of Ottawa's Research Ethics Board (S-06-18-428). Participation was voluntary, and participants provided written informed consent and were assured of confidentiality of the information collected in the study. To protect identities, pseudonyms were used in transcripts. No compensation was provided.

## Results

### Overview of Respondents

**Figure 1.**
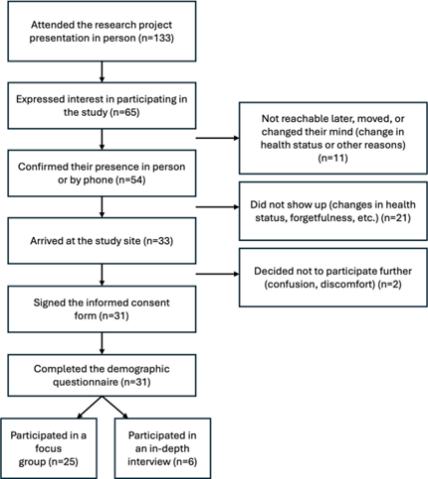
Recruitment process: This flowchart illustrates the recruitment process for the study, detailing the number of participants at each stage. Of the 133 project presentation attendees, 65 expressed interest, 54 confirmed participation, and 33 arrived at the study site. Ultimately, 31 signed consent and completed the demographic questionnaire, with 25 joining a focus group and 6 participating in an in-depth interview. Drop-off reasons included inaccessibility, health changes, and discomfort.

A total of 133 individuals attended the initial research presentation, with 65 expressing interest. Of these 65, 54 (85.7%) confirmed participation, but some later withdrew due to personal or health reasons or became unreachable. Ultimately, 31 participants completed the study (25 in focus groups and 6 in in-depth interviews; Figure 1).

The study included 31 participants from various care settings: SAFE, LTC, and ILA (Table 1). Of the 6 interviewees, 5 were from SAFE and 1 from LTC. Age varied by living environment, with LTC participants being the oldest. Gender representation was balanced, with 45.2% male and 54.8% female. A significant portion (42%) had university-level education. Most were widowed or single, lived alone (64.5%), and had chronic conditions, with 45.2% reporting comorbidities. Despite health issues, 67.8% rated their health as good or better.

**Table 1.** Participants’ characteristics.

Characteristics	Focus groups (n=25)			Interviews (n=6)	Total (n=31)
	SAFE^a^ unit (n=7)	Long-term care (n=7)	Independent living (n=11)		
Age, years, median (range)	78 (65-87)	94 (87-99)	89 (72-93)	78 (65-97)	87 (65-99)
Gender, n (%)					
Male	1 (14.3)	5 (71.4)	5 (45.5)	3 (50)	14 (45.2)
Female	6 (85.7)	2 (28.6)	6 (54.5)	3 (50)	17 (54.8)
Education, n (%)					
Nonuniversity education^b^	5 (71.4)	2 (28.6)	5 (45.5)	3 (50)	15 (48.4)
University-level education^c^	2 (28.6)	5 (71.4)	6 (54.5)	3 (50)	16 (51.6)
Marital status, n (%)					
Married/partnered	3 (42.9)	—^d^	3 (27.3)	2 (33.3)	8 (25.8)
Widowed	3 (42.9)	6 (85.7)	6 (54.5)	3 (50)	18 (58.1)
Single/divorced/separated	1 (14.3)	1 (14.3)	2 (18.2)	1 (16.7)	5 (16.1)
Living situation, n (%)					
Lives alone	4 (57.1)	7 (100)	6 (54.5)	3 (50)	20 (64.5)
Lives with partner	2 (28.6)	—	3 (27.3)	2 (33.3)	7 (22.6)
Other	1 (14.3)	—	2 (18.2)	1 (16.7)	4 (12.9)
Self-reported health, n (%)					
Good to excellent	4 (57.2)	4 (57.1)	9 (81.9)	4 (66.7)	21 (67.8)
Fair to poor	3 (42.8)	3 (42.9)	2 (18.1)	2 (33.3)	10 (32.2)
Chronic illness, n (%)					
None	—	—	3 (27.3)	—	3 (9.7)
Single condition	2 (28.6)	5 (71.4)	5 (45.5)	2 (33.3)	14 (45.2)
Comorbidities	5 (71.4)	2 (28.6)	3 (27.3)	4 (66.7)	14 (45.2)

a SAFE: subacute unit for frail elderly.

b Includes high school, trade, college, and other nonuniversity education.

c Includes participants with bachelor’s or graduate degrees.

d —: not applicable.

### Key Findings

**Figure 2.**
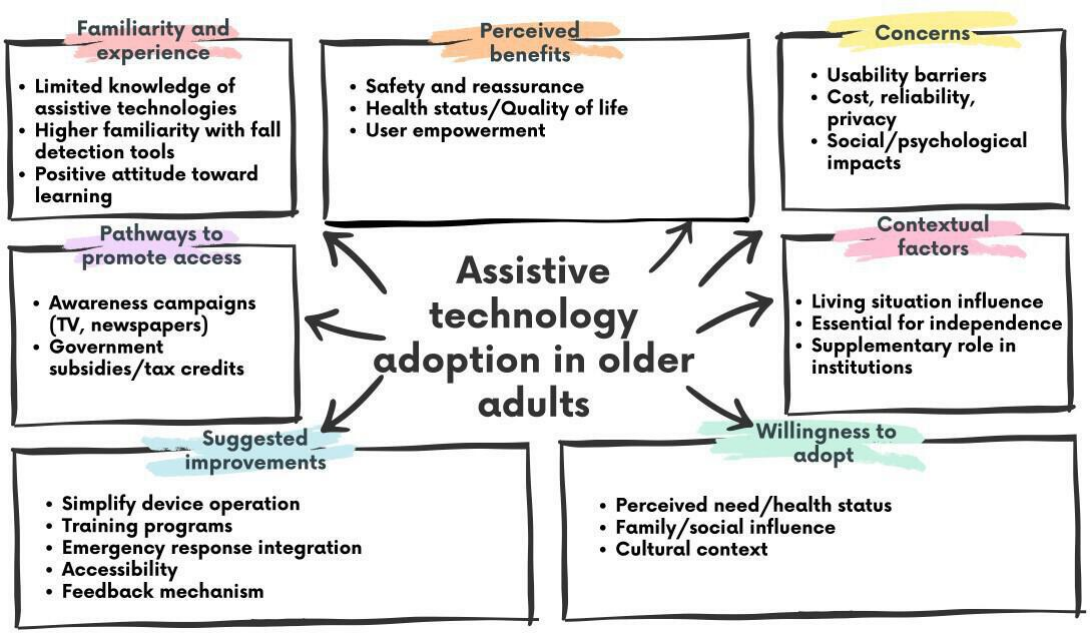
Key themes and subthemes: This diagram highlights factors influencing assistive technology adoption in older adults, including familiarity, perceived benefits, concerns, contextual factors, willingness to adopt, pathways to promote access, and suggested improvements.

We identified 7 overarching themes from the thematic analysis conducted on the data generated from the focus groups and interviews, which are summarized in Figure 2.

#### Theme 1: Familiarity and Experience With Technology

**Figure 3.**
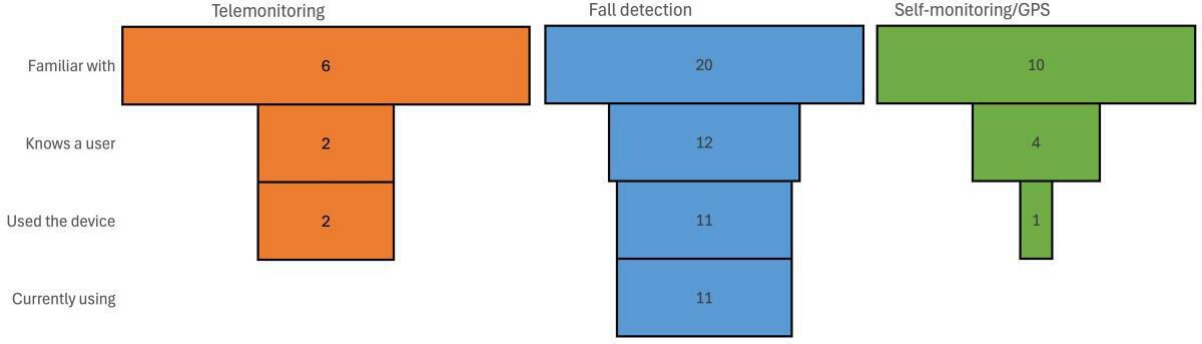
Familiarity with and use of assistive technologies: This chart shows participants’ familiarity and engagement with telemonitoring, fall detection, and self-monitoring devices. Fall detection devices had the highest familiarity and usage, whereas self-monitoring devices had moderate awareness but low current usage. Telemonitoring had the lowest familiarity and minimal adoption.

Most participants had limited prior knowledge of ATs. While many of them followed the news through newspapers and television, they knew little about technologies that could support them. Some participants were eager to learn more, whereas others remained indifferent. As shown in Figure 3, familiarity was highest for fall detection (n=20), followed by self-monitoring/GPS (n=10), and lowest for telemonitoring (n=6). We observed a similar pattern for other measures of exposure, such as knowing a user or having used the device themselves. These differences in baseline familiarity appeared to influence openness and perceived relevance during group discussions. Despite limited experience with these investigated ATs, older adults expressed a positive attitude toward them, for example, “I’m glad to learn there are things like this happening” and “Knowing about these devices motivates us to continue living and maintaining our independence.”

#### Theme 2: Perceived Benefits

The participants identified 3 key benefits of ATs presented below.

##### Safety and Reassurance

Participants commonly perceived that all 3 ATs can reassure users and their families by enabling timely emergency interventions, although they had not personally used these technologies. They valued telemonitoring for its ability to detect abnormalities early and provide prompt feedback. Self-monitoring tools were praised for tracking users’ locations, helping families ensure the safety of loved ones who might become disoriented or lost. Fall detection technology was seen as lifesaving, with 1 participant stating, “Using a fall detector gives the family peace of mind, knowing that their loved one won’t be found on the floor hours later.”

##### Health Status and Quality of Life

ATs were seen as beneficial for older adults’ health by improving chronic disease management (telemonitoring), enabling timely interventions (fall detection), and encouraging active lifestyles (self-monitoring). They also support independence, particularly for those living at home. As 1 participant noted, “This could extend the time they can live at home and do certain activities,” instead of being institutionalized.

##### User Empowerment

Participants felt that telemonitoring’s immediate feedback from health care professionals fostered a sense of control and informed decision-making. As 1 participant shared, “If she says in the morning, ‘I feel this way,’ and the [healthcare professional] responds within an hour, she will know what to do.”

### Theme 3: Concerns

Despite the perceived benefits, participants raised several key concerns regarding the use of ATs.

#### Usability

Participants highlighted physical, cognitive, and educational barriers that could limit ATs’ adoption and effective use. As 1 respondent noted, “Telemonitoring can only be used by someone who can use it manually, but what if you can’t use your hands?,*”* raising concerns that some users may struggle with device operation.

#### Cost

Many participants saw high expenses as a barrier to widespread adoption and equitable access. As 1 respondent observed, “I see a huge divide between people who can afford this type of technologies and those who can’t.” This concern about cost reflects broader patterns of digital exclusion among older adults, as noted in recent reviews on health technology disparities [18,65]. Such perceptions may also compound age-related income constraints, underscoring the need for subsidized or publicly funded solutions.

#### Reliability and Response Time

Participants were concerned about the risk of incorrect or delayed information and the ability to act on it. One respondent questioned, “What happens if there’s a problem? Will someone be alerted immediately? The response time matters a lot.” The effectiveness of telemonitoring depends not only on the technology but also on the professionals overseeing it. Some were skeptical about whether professionals actively monitored the data, with 1 participant indicating, “It feels like they don’t actively monitor the device. For me, it doesn’t seem worthwhile,” suggesting potential trust-related concerns.

#### Privacy and Data Security

Most participants were concerned about potential breaches of sensitive information or data being shared with third parties, such as insurers. One respondent also feared that ATs could increase vulnerability to theft, stating, “It could very easily enable someone with malicious intention to access the system and discover that you are not at home.*”*

#### Social and Psychological Impacts

Participants expressed concerns about the psychological effects of ATs’ use, including feelings of dependency and loss of dignity. Many saw these devices as symbols of weakness or reliance on others, potentially impacting self-esteem and personal pride. As one participant noted, "You feel like you don’t want to be the person who has to wear that, and yet, in reality, you have to.*”* Participants’ emphasis on the psychological burden of using fall detectors echoes findings from Köttl et al [66], where older adults internalized negative stereotypes linked to assistive device use, framing them as “symbols of decline.” Our findings further confirm that emotional readiness is just as critical as functional need in ATs’ adoption decisions.

#### Acceptance and Familiarity

The novelty of ATs contributed to hesitation and skepticism among participants. Some were cautious about adopting unfamiliar devices, even when aware of their benefits. As 1 participant remarked, “People might not trust new devices. Anything new is hard to trust, isn’t it?”

### Theme 4: Suggested Improvements

Participants recommended designing *simpler, user-friendly* devices, as many older adults may struggle with complex technology. They stressed the importance of *training and orientation*, with 1 participant noting, “You can’t just hand someone something and leave them to figure it out. There needs to be training or orientation.” To enhance emergency response, respondents suggested integrating location-tracking with fall detection devices to ensure faster assistance. As 1 participant proposed, “If they press an alarm, we should be able to locate them, no matter where they are.*”*

Accessibility was another key concern, with many advocating for features that accommodate physical and cognitive limitations, “like using large buttons or voice commands”*.* Reminder systems (for forgetfulness), expanded coverage, and wearable options were also highlighted. One participant remarked, “If they want to change the world, these systems should be everywhere, not just in nursing homes.*”* Finally, participants valued feedback features to confirm help was on the way. As 1 participant stated, “...if it could notify you that your message was sent and someone is coming, that would be reassuring.*”*

### Theme 5: Contextual Factors Influencing Use

Perceived usefulness varied by living situation. For older adults living independently, all 3 ATs were viewed as essential for safety, health, and independence. As 1 participant explained, “It’s good if you’re not in a critical state requiring hospitalization, but you have medical conditions that need monitoring, which can be done remotely.*”*

In institutional settings, these technologies were seen as supplementary to existing care. With professional staff available around the clock, some participants saw telemonitoring and fall detection as redundant. However, others still found value in them, with 1 participant noting, “Even in an institution, I could fall in my room, and staff might not immediately know”

### Theme 6: Pathways to Promote Awareness/Access

Awareness campaigns using accessible media were suggested: “Television and newspapers would be good ways to inform people about these options.*”* Government support (ie, subsidies, need-based assistance, and tax credits) to offset costs was discussed as critical. While such support does not currently exist for ATs, participants referenced similar programs in Ontario that subsidize other physician-prescribed medical equipment, such as continuous positive airway pressure machines.

### Theme 7: Willingness to Adopt/Use ATs

Many participants initially expressed enthusiasm toward ATs, “This is fantastic!,” but this did not necessarily translate into a willingness to adopt them: “My diabetes isn’t severe enough right now!*”* or “You can lead a horse to water, but you can’t make it drink.” Their willingness to use these technologies depended on several key factors.

#### Perceived Need and Health Status

Many participants indicated that their willingness to use ATs would increase if their health declined. As 1 participant noted, “I think you have to experience a fall to scare you enough to jump into it faster.” Others recognized the potential benefits but felt no immediate need due to stable health conditions.

#### Family and Social Influence

Family encouragement played a crucial role in AT adoption. Some participants described how their families insisted on using these technologies for peace of mind, even when they were hesitant. As 1 participant shared, “My kids keep pushing me to try it [fall detection bracelet] because it makes them feel better about my safety.*”*

#### Health Care Recommendations

Guidance from health care professionals significantly influenced ATs’ adoption. As 1 participant stated, “If my doctor recommended it, I’d definitely consider it,*”* highlighting the trust placed in medical advice.

#### Ease of Use and Design

Participants stressed that ease of use was crucial in their decision to adopt ATs. Lightweight, user-friendly, and accessible devices were more appealing, whereas complicated or impractical designs discouraged interest. In addition, limited awareness of how these technologies functioned created barriers to adoption.

#### Emotional and Psychological Concerns

Participants expressed mixed feelings about being monitored, with some finding it intrusive, whereas others appreciated the security it provided. As 1 participant stated, “It’s a bit unsettling to know you’re always being watched, but it’s also comforting to know help is there if you need it.*”*

#### Cultural and Situational Context

Social norms and perceived popularity influenced interest in AT adoption. As 1 participant noted, “If other people have it (telemonitoring) and I don’t, I feel disadvantaged.” ATs were more appealing to those living alone or in rural areas, whereas individuals in assisted living homes or with regular care access showed less interest.

## Discussion

### Principal Findings

This study explored older adults’ perceptions of 3 ATs, namely, telemonitoring systems, fall detection devices, and self-tracking tools. By combining focus groups and in-depth interviews, we gathered nuanced perspectives from individuals with varied health statuses and living environments. This design helped uncover both attitudinal and contextual factors shaping AT acceptance, contributing to an enriched understanding of older adults’ engagement with technology.

### Perceived Benefits and the Role of Identity

Participants consistently acknowledged the potential of ATs to enhance safety, independence, and chronic disease management. These findings align with the literature, suggesting that older adults are not inherently resistant to technology, but that they appraise its value through the lens of personal relevance and life context [67-71]. Importantly, this study highlights a tension between recognition of benefits and personal adoption—participants often deemed ATs useful “for others,” but not necessary for themselves.

This ambivalence may reflect deeper psychological dynamics. Drawing on Socioemotional Selectivity Theory [72], many participants appeared to prioritize emotional satisfaction and autonomy in the present over future-oriented planning. Similarly, internalized ageism, the absorption of negative stereotypes about aging, was evident in language that framed ATs as symbols of frailty or decline. Participants’ reluctance to adopt fall detection devices, in particular, often stemmed from concerns about stigma or diminished self-image. These reactions echo prior findings that older adults resist technologies perceived to signal vulnerability [66], underscoring the importance of identity preservation in technology acceptance.

### Barriers to Adoption: Beyond Attitudes

Despite generally favorable attitudes, several barriers limited participants’ readiness to adopt ATs. Cost emerged as a prominent concern, even in Canada’s publicly funded health care context. Consistent with UTAUT2 [73-76], where “price value” influences behavioral intention, participants were highly sensitive to the cost–benefit tradeoff. For many, high device costs were incompatible with fixed or limited incomes. This highlights the need for policy interventions, such as subsidies or expanded Assistive Devices Programs, to enhance financial accessibility.

Usability was another critical barrier. Participants emphasized the importance of accessible, intuitive designs, particularly given potential sensory, physical, or cognitive impairments. These findings reinforce prior research linking effort expectancy (ease of use) to adoption intent [77,78]. Participants suggested adaptive features (eg, voice commands and large buttons) and emphasized the value of hands-on training and orientation sessions. These recommendations support calls for participatory design approaches that incorporate older adults’ perspectives into AT development [7,78,79].

Although privacy concerns were noted, they were generally outweighed by perceived safety and health benefits. Still, these concerns merit further investigation. Recent extensions of UTAUT have treated privacy either as an independent predictor [80] or a moderator [81], but its role in older adult populations remains underexplored. Future research should test whether privacy functions differently in this demographic, particularly with respect to self-tracking and location-based technologies.

### Willingness Versus Readiness: A Critical Distinction

This study also underscores a conceptual distinction between willingness (attitudinal openness) and readiness (practical ability to act). While participants expressed interest in ATs, this rarely translated into immediate adoption. Readiness appeared contingent on several enabling conditions: digital literacy, health triggers (eg, experiencing a fall), social support, and recommendations from trusted others. This distinction is rarely emphasized in AT literature but is essential for tailoring interventions. Addressing readiness gaps through skills training, caregiver involvement, and experiential exposure to ATs may be just as important as shifting attitudes.

### The Influence of Experience and Social Context

Experience with ATs emerged as a powerful enabler. Participants with prior exposure demonstrated higher confidence, clearer expectations, and greater openness to ongoing use. This finding supports evidence from diffusion studies that familiarity reduces perceived complexity and increases trialability [82-84]. Conversely, nonusers often failed to recognize personal relevance, even when their health status suggested potential benefit.

Here, the diffusion of innovation framework [85] offers a useful lens. Health professionals, especially in institutional settings, can serve as gatekeepers, opinion leaders, and change agents. For example, physicians and occupational therapists can assess individual needs, demystify technologies, and provide credible endorsements, thereby influencing both perceived usefulness and social norms [86-88]. Our findings reinforce the importance of leveraging these roles, especially within LTC contexts where allied health professionals routinely assess functional abilities. Promoting ATs through routine care conferences, orientation sessions, and interdisciplinary planning can normalize their use and personalize recommendations.

### Implications for Design, Policy, and Practice

Bridging the gap between positive attitudes and actual adoption of ATs requires multipronged strategies that target both systemic and individual-level barriers. Participants emphasized the importance of adaptive, inclusive designs that accommodate cognitive, sensory, and physical limitations. Features such as voice activation, tactile interfaces, and customizable visual displays (eg, font size and button shape) were seen as particularly valuable for enhancing usability. In line with earlier research [7,39,89,90], these design features can help ensure technologies are accessible to a wider range of older adults.

Participants also stressed the importance of training and support. Many felt that simply distributing a device without orientation would be ineffective. Suggestions included hands-on demonstrations, step-by-step tutorials, and reinforcement opportunities through follow-up sessions. For example, in LTC settings, technology vendors could be invited to host demonstration days with staff, residents, and caregivers. For community-dwelling older adults, device trials combined with user support, potentially involving caregivers or trained volunteers, could enhance experiential learning and encourage integration into daily life.

Education and outreach are equally important. Participants advocated for awareness campaigns using accessible media, such as television and newspapers, to reach broader audiences. These campaigns should emphasize the empowering aspects of ATs, helping reframe them as tools for maintaining independence rather than markers of decline. This is particularly critical in combating internalized ageism and reshaping social narratives around aging and technology.

Health care providers, especially physicians, nurses, and allied health professionals, play a pivotal role in recommending and normalizing AT use. Their trusted relationships with older adults position them as effective intermediaries for personalized advice and confidence-building. Strengthening provider involvement through continuing education, clinical guidelines, and structured patient discussions can help increase uptake. In LTC, allied health professionals such as occupational and physiotherapists are well-positioned to incorporate AT assessments into routine care planning. Opportunities for integration include resident orientation, care conferences, and interdisciplinary team meetings. These touchpoints offer natural opportunities to introduce ATs aligned with residents’ values, needs, and preferences [91].

Finally, policy interventions are also essential. Public reimbursement schemes, such as Ontario’s Assistive Devices Program, could be expanded to include a broader array of ATs and cover maintenance or replacement costs. Such support would help address cost-related concerns, one of the most frequently cited barriers to adoption, and ensure equitable access. Policymakers may also consider embedding AT content into the curricula of health care training programs, ensuring that future providers across disciplines are equipped to assess, recommend, and support AT use among older adults.

### Limitations and Avenues for Future Research

This study has limitations that must be acknowledged. First, given its exploratory nature, we used an inductive approach through focus groups and interviews to capture older adults’ experiences without restricting responses to predefined frameworks (eg, TAM, UTAUT). This approach enabled novel insights to emerge, although future studies could build on our findings using established models to assess generalizability and validate results in other contexts.

Second, recruitment was limited to individuals with sufficient cognitive abilities to provide informed consent, excluding those with significant impairments. Physical and sensory limitations, such as hearing or vision issues, sometimes disrupted group discussions and required clarification. In some cases, small group sizes—due to cancellations or misunderstandings—reduced discussion depth. However, in-depth interviews helped address these gaps by capturing additional perspectives. Time constraints also limited exploration of some topics during focus groups, although these were partially mitigated by the interviews.

Third, participants viewed brand-neutral video demonstrations to facilitate discussion, but these may have shaped perceptions based on how the technologies were presented. While the videos offered general overviews, they did not depict specific devices, limiting discussion of their particularities. In addition, themes such as privacy, cost, and emergency response were included in the interview guide, potentially influencing responses. For confidentiality, quotes were not linked to participants’ identities or care settings, preventing comparative analysis across environments.

Despite these limitations, the study addressed several gaps in the extant literature. We explored 3 types of ATs relevant to diverse care needs and included 31 participants from LTC, SAFE, and independent living settings, offering broader insights than prior studies with smaller or more homogeneous samples. Unlike studies relying on a single method [83,92-98], our combined use of focus groups and interviews allowed for a richer, more nuanced understanding. Still, we were unable to include participants with cognitive impairment. Future research should address this by involving informal caregivers, who play a key role in decision-making and technology use. Additional research is also needed to explore AT adoption among individuals with greater physical or cognitive limitations. Quantitative studies examining perceived need, prior experience, and external influences (eg, clinician recommendations) could inform strategies to support wider adoption.

### Conclusions

Promoting the use of health ATs among older adults requires a focus on improving accessibility, usability, and awareness. This research adds to the growing body of literature on older adults’ perspectives on health ATs by offering insights into their attitudes, barriers, and facilitators of adoption. While older adults recognize the benefits of telemonitoring systems, fall detection technology, and self-tracking devices, adoption remains limited and is influenced by factors such as perceived personal need, prior experience, and accessibility. Future efforts should focus on creating adaptive designs, offering experiential opportunities, and implementing accessible pricing models to enhance the adoption of health ATs and maximize their potential to support aging populations.

## Abbreviations

AT: assistive technology
ILA: independent living apartments
LTC: long-term care
SAFE: subacute unit for frail elderly
STAM: Senior Technology Acceptance Model
UTAUT: Unified Theory of Acceptance and Use of Technology
